# Ultrasound‐guided sciatic nerve block at the midthigh level in a porcine model: A descriptive study

**DOI:** 10.1002/vms3.265

**Published:** 2020-04-12

**Authors:** Mi Geum Lee, Sung Uk Choi, Jae Kwan Lim, Mee Ju Lee, Ji Su Hong, Mi Ok Baek, Seung Zhoo Yoon, Hee Yeon Park, Hyeon Ju Shin

**Affiliations:** ^1^ Department of Anesthesiology and Pain medicine Gachon University College of Medicine Incheon South Korea; ^2^ Department of Anesthesiology and Pain medicine Korea University College of Medicine Seoul South Korea; ^3^ Korea Artificial Organ Center Korea University College of Medicine Seoul South Korea

**Keywords:** hindlimb surgery, Midthigh, pig, sciatic nerve block, ultrasound

## Abstract

**Background and Objective:**

There are a growing number of porcine models being used for orthopaedic experiments for human beings. Therefore, pain management of those research pigs using ultrasound (US)‐guided nerve block can be usefully performed. The aim of this study is to determine optimal US approaches for accessing and localizing the sciatic nerve at the midthigh level, a relevant block site for hindlimb surgery in female Yorkshire pigs.

**Methods:**

As a first step, we dissected the intubated, blood‐washed out pigs (*n* = 3) and confirmed the anatomical position of the sciatic nerve at midthigh level. After dissection, we found the sciatic nerve, connected with nerve stimulator, and checked the dorsiflexion or plantar flexion of the hindlimb. We matched the sciatic nerve location with the US image. After the pigs were euthanized, the neural structures of the sciatic nerve were confirmed by histological examination with H&E staining. In second step, a main US‐guided sciatic nerve block study was done in the intubated, live pigs (*n* = 8) based on the above study.

**Results:**

In lateral position, the effective US‐guided nerve block site was about 6 cm from the patella crease level; immediately proximal to the bifurcation of the sciatic nerve into the tibial nerve and common peroneal nerve. The distal femur was selected as the landmark. There were no vessels or other nerves surrounding the sciatic nerve. The needle‐tip was positioned less than 1 cm lateral from the distal femur and about 2 cm deep to skin. ‘Donut sign’ in US images was confirmed in all 16 nerves.

**Conclusions:**

Midthigh level sciatic nerve is located superficially, which enables nerve block to be easily performed using US. This is the first study to describe midthigh sciatic nerve block in the lateral position under US guidance in a porcine model from a clinical perspective.

## Graphical abstract

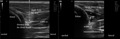

## 1 INTRODUCTION

A large animal model has a more similar anatomy and weight bearing to human joints than rat and rabbit model; thus, a growing number of porcine models have been used for orthopaedic experiments for human beings (Goetz et al., 2015; Kotsougiani et al., 2019; Visser et al., 2019). But effective pain management of those research pigs seems to be overlooked by orthopaedic researchers, and a nerve block‐related study using pig model is scarce contrary to dogs and calves (Benigni, Corr, & Lamb, 2007; Costa‐Farré, Blanch, Cruz, & Franch, 2011; Echeverry et al., 2010; Re, Blanco‐Murcia, Vellaescusa, Gaspar, & Gómez de Segura, 2014; Royal et al., 2013).

The use of nerve block techniques allows an effective analgesia in veterinary patients along with systemic opioids or NSAIDs because it can reduce the dosage of the analgesic drugs (Royal et al., 2013).

In this study, we attempted an ultrasound (US)‐guided sciatic nerve block at midthigh level in a porcine model. We used an US‐guided nerve block, and real‐time visualization of spreading of the local anaesthetic near the target nerve allowing us to use reduced drug volume, and to have faster onset of blockade, and to increase the success rate of the nerve block compared to using surface anatomical landmarks or nerve stimulator only (Costa‐Farré et al., 2011; Echeverry et al., 2010).

US‐guided sciatic nerve block has been used for anesthesia and analgesia of the hindlimb, blocking at gluteal level (Costa‐Farré et al., 2011; Gurney & Leece, 2014; Royal et al., 2013). However, when surgery is required in the distal part of the pelvic limbs, block at midthigh level is sufficient (Echeverry et al., 2010; Re et al., 2014). In humans, US‐guided sciatic nerve block has frequently been performed at the popliteal level for foot and ankle surgery (Barrington, Lai, Briggs, Ivanusic, & Gledhill, 2008; Karaarslan et al., 2016; Triadó et al., 2004). In US technique, easy accession and simple needling are important in preventing nerve injuries and increasing the block success rate (Echeverry et al., 2010; Re et al., 2014; Triadó et al., 2004).

The aim of the present study is to define a method that facilitates access to the midthigh sciatic nerve via anatomical study in blood‐washed out pigs, and to describe the clinical applications of sciatic nerve block in live pigs by needle injection under US guidance from a clinical perspective.

## 2 MATERIALS AND METHODS

### 2.1 Animals

This study was approved by the Korea University Institutional Animal Care and Use Committee (KUIACUC‐2015‐17). Eleven female Yorkshire pigs weighing 40.5 ± 0.76 kg and aged 11 ~ 12 weeks were used in this study. The pigs were provided as ‘bacon pigs’ (GP Farms, Orient Bio, South Korea) and adapted to the Animal Facility at the Korea University for 2 weeks prior to study. Animals were housed in raised individual pens (2 × 2 m), fed a maintenance diet (pig fodder, Purina Feeds Ltd.), and permitted free access to tap water in a temperature controlled environment (23 ± 2.3°C) under a 12:12 hr light/dark cycle. The pigs were fasted for 24 hr preoperatively. The anaesthetic medication consisted of xylazine (Rompun^®^, 1 mg/kg) and zolazepam/tiletamine (Zoletil^®^, 7 mg/kg) was administered intramuscularly. Animals were then placed on surgical tables in a supine position with neck extended and monitored by pulse oximetry and electrocardiography. A catheter was inserted into the auricular vein, and during anesthesia, Ringer's lactate solution was administered at 5 ml kg^−1^ h^−1^.

Pigs were intubated with a standard cuffed endotracheal tube (ETT; 6 mm internal diameter; Portex^®^, Smith‐Medical), and the cuff was inflated with 15 ml of air. Correct ETT position was confirmed by direct visual examination by laryngoscopy and bilateral chest auscultation. Anesthesia was maintained with enflurane at an end‐tidal concentration of 1.5%. Fresh gas flow was maintained at 4 L/min with FiO_2_ of 0.5. Tidal volume and respiratory rate were set at 8 ml/kg and 15 breaths/min, respectively.

### 2.2 Anatomical study in blood‐washed out porcine model

Three Yorkshire pigs were used in this study. The anatomical position of the sciatic nerve in the designated hindlimb was determined according to an atlas of swine anatomy (Peter, 2012). This study was performed immediately after an experimental cardiovascular trial, removing all blood of the pigs in the intubating state, and the response of nerve stimulating was alive.

**FIGURE 1:**
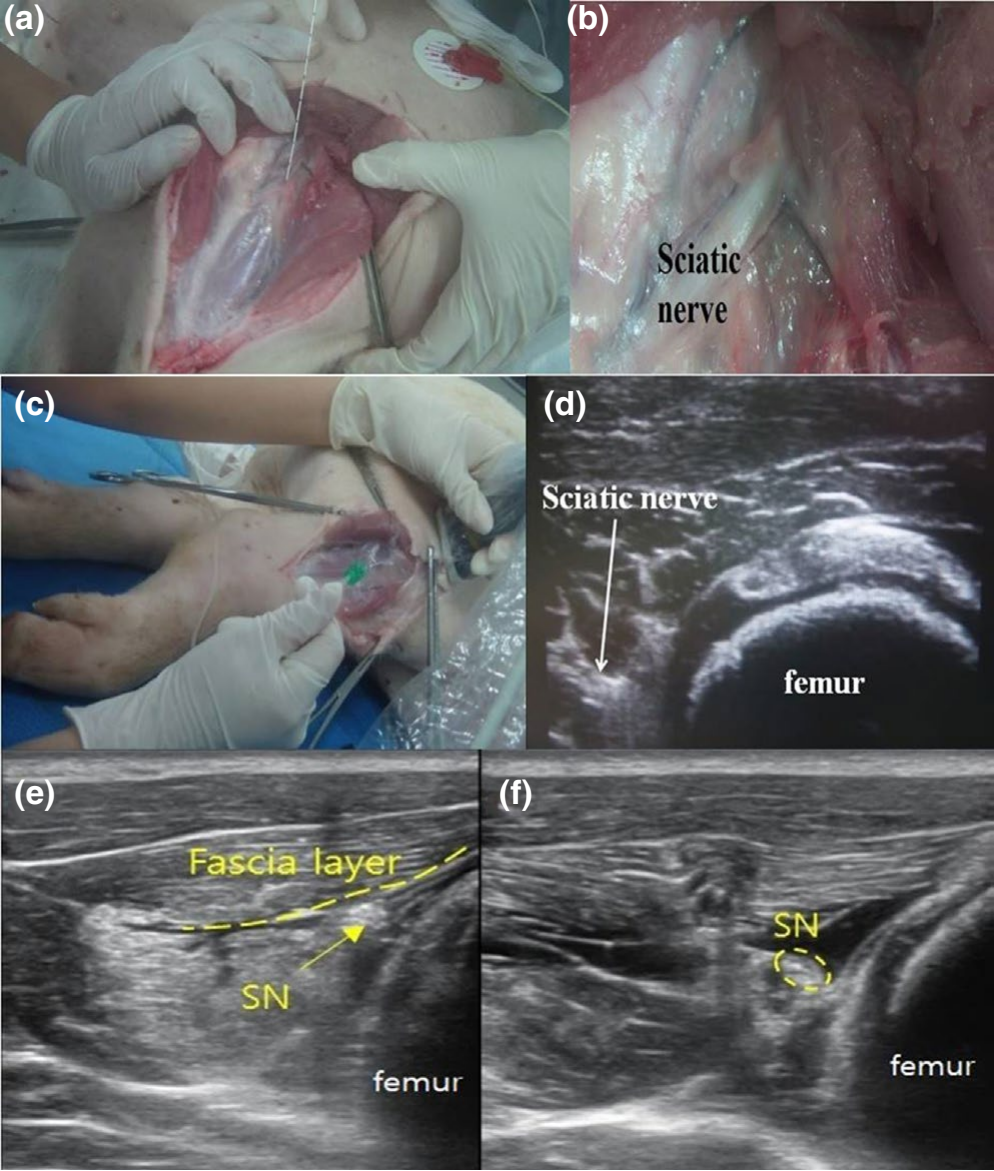
Anatomical study of the sciatic nerve in a blood‐washed out porcine model at the midthigh level. (a) After dissection, the sciatic nerve connected to the nerve stimulating needle (b) an enlarged image of the sciatic nerve (c) tracing with US probe perpendicularly above the skin over the dissected structures (d) matching the sciatic nerve position with the US short‐axis image (e) pre‐injected sciatic nerve and attached fascia layer (f) post‐injected sciatic nerve which is separated with fascia layer. SN for the sciatic nerve

In three pigs, skin on the lateral aspect of the midthigh from the midline between the greater trochanter of the femur and the ischial tuberosity was incised along the boundary of the biceps femoris muscle distally; other muscles were left intact. The biceps femoris was then reflected towards the greater trochanter and the sciatic nerve identified (Figure 1a and b).

A 22 gauge, 120 mm needle (Stimuplex A^®^, B. Braun Medical Inc.) with connecting to a nerve stimulator (B.Braun Medical Inc.) was inserted into the perineural space of the sciatic nerve at 0.5 mA (2 Hz), and the sciatic nerve was confirmed by dorsiflexion induced by the common peroneal nerve or plantar flexion by the tibial nerve.

The skin and muscle was re‐covered with the needle which was still positioned in the perineural space of the sciatic nerve. A 6–13 MHz linear US transducer (ECUBE9^®^ Alpinion) was placed perpendicularly just above the skin. And we found the site in which the sciatic nerve can be seen immediately proximal to the bifurcation into tibial and common peroneal nerves by scanning the probe in cephalad or caudad direction to match its anatomical position with the US short‐axis image (Figure 1c and d).

**FIGURE 2:**
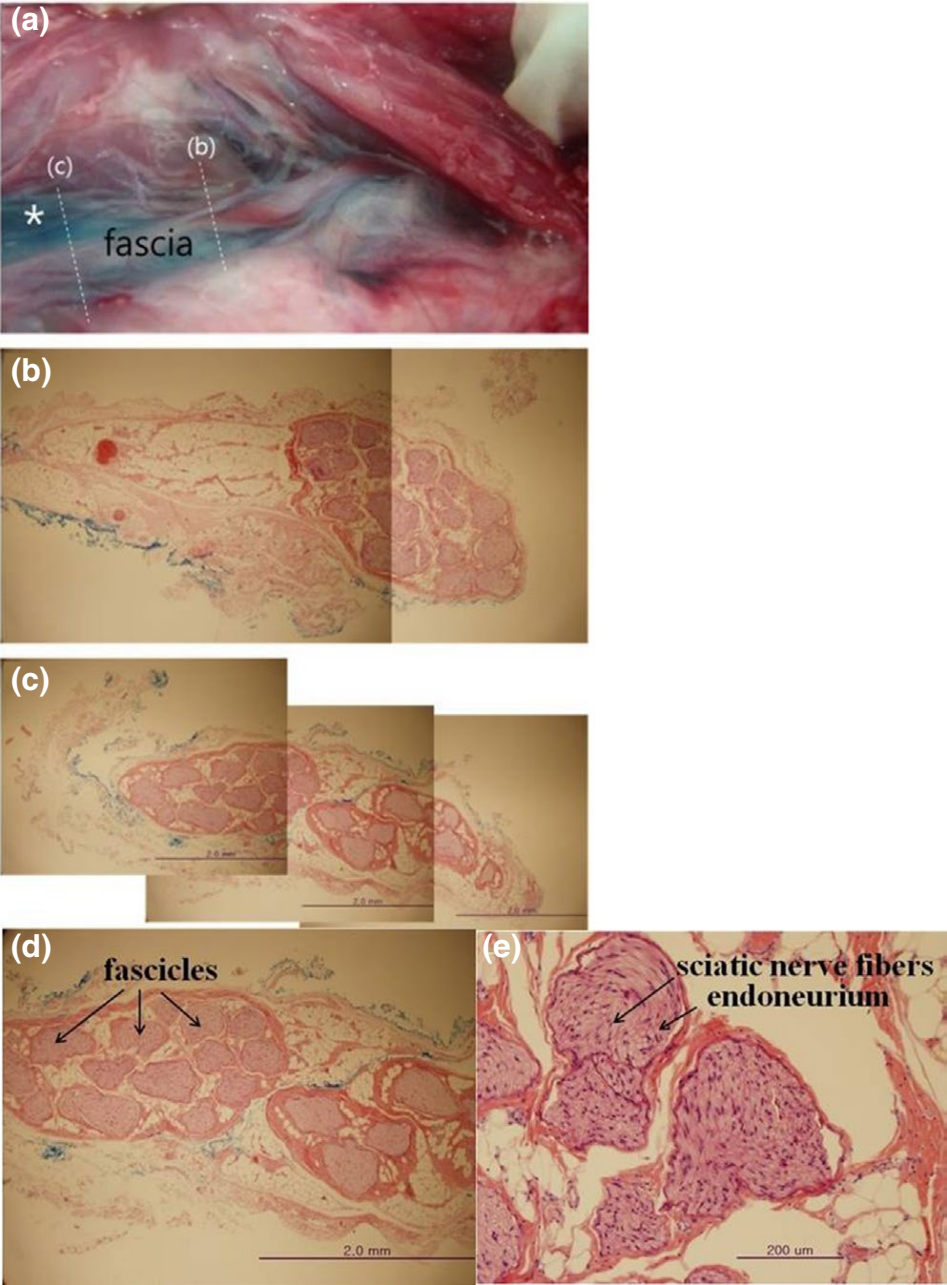
The histopathological study of the sciatic nerve by the H&E staining. (a) post‐staining perineural space of sciatic nerve (*) (b) cross section of the sciatic nerve at the site of ‘before‐bifurcation’ (c) cross section of the sciatic nerve at the site of ‘post‐bifurcation’ (d) magnification of figure c in which methylene blue is outside the external epineurium (e) magnification of figure d in which the gaps between the nerve bundles expanded by local anaesthetics with no inflammatory finding

Lidocaine (LDC; 1.5%, 20 ml) mixed with methylene blue was injected via the needle into the perineural space of the sciatic nerve. After we confirmed circumferential spread of LDC around the sciatic nerve in US short‐axis view (Figure 1e and f), the pig was euthanized (Voelckel et al., 2005). The sciatic nerve was carefully freed from surrounding tissue and removed for microscopic examination. Histological examination of dissected sciatic nerves (pre‐branching site and post‐branching site) was performed by H&E staining to confirm their identities (Figure 2).

### 2.3 Main US‐guided nerve blockade in live porcine model

Sixteen sciatic nerves of eight pigs were subjected to this main nerve block study. Each intubated pig was positioned laterally on a surgical table.

**FIGURE 3:**
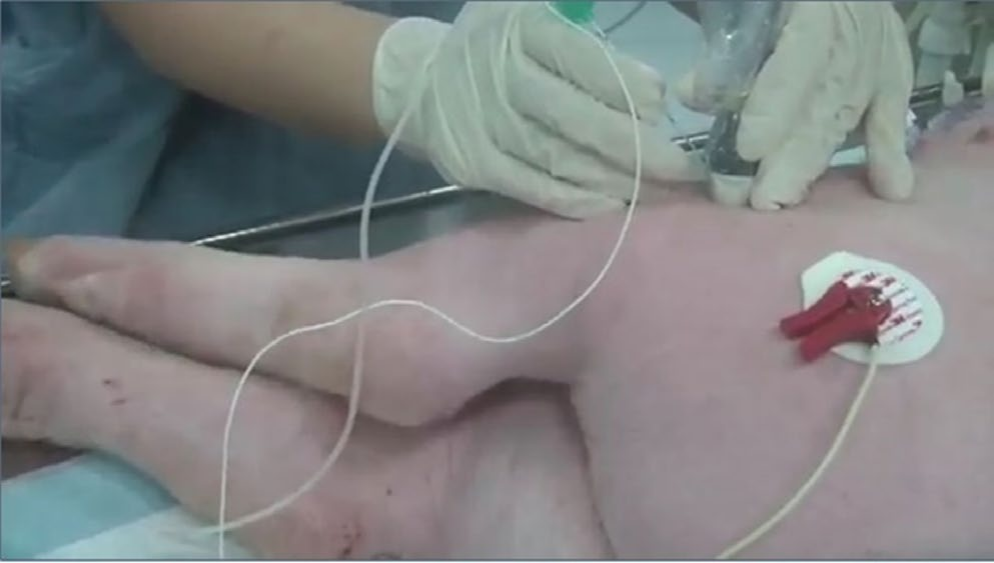
Ultrasound transducer position for midthigh sciatic nerve block, about 6 cm above the popliteal crease

First, we placed the US probe perpendicularly between the greater trochanter of the femur and the ischial tuberosity to follow the sciatic nerve. We moved the probe distally to the bifurcation into the tibial and common peroneal nerves until patella crease level, and found the block site based on the above anatomical study (Figure 3).

We used US and nerve stimulator simultaneously with out‐of‐plane approach in which the needle was seen in as a hyperechoic ‘dot’ on the US image (Costa‐Farré et al., 2011). Once the needle was precisely inserted into the perineural space (based on direct needle tip in the scanning plane and tissue displacement), a nerve stimulator was applied at 0.5 mA (2 Hz). The intensity was increased or decreased until evident applicable muscle contraction (Figure 3; Video S1) and we confirmed the proximity of the needle tip to the nerve at 0.3–0.5 mA (Costa‐Farré et al., 2011).

**FIGURE 4:**
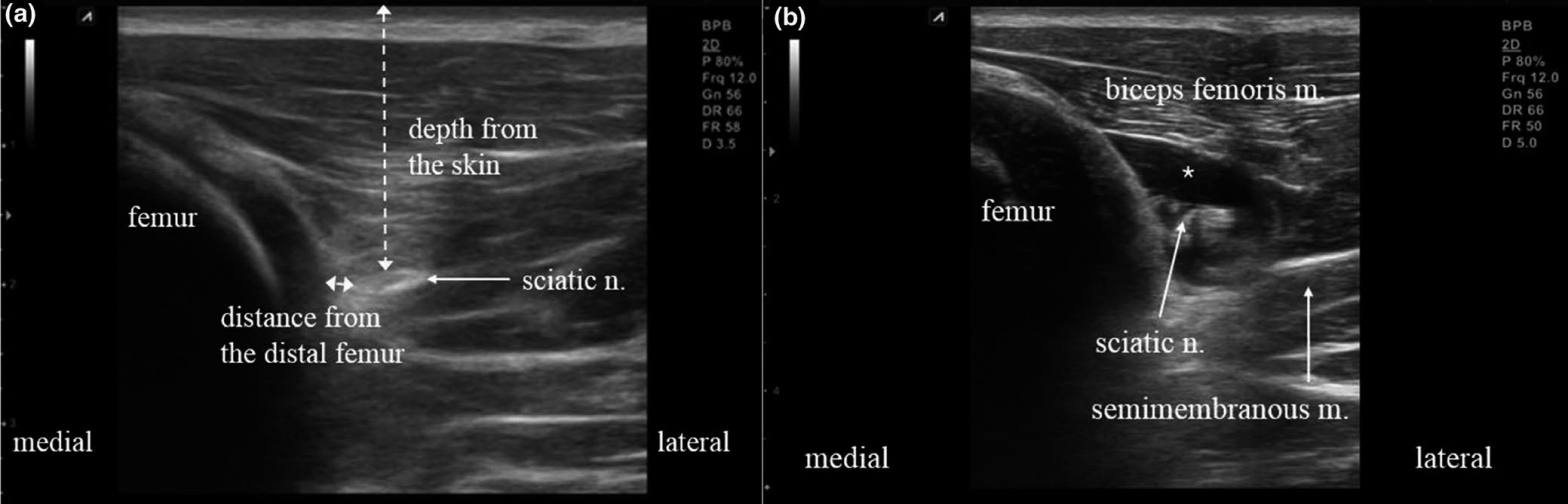
Ultrasound short‐axis images of pre‐ and post‐local anaesthetic injections in the sciatic nerve. (a) pre‐injection sciatic nerve location defined by measuring distances from skin (broken line) and the distal femur (solid line). (b) After injection sciatic nerve which is clearly separated from surrounding tissues (donut sign). *: local anaesthetic

After that, LDC was injected after negative aspiration into the perineural space of the sciatic nerve; muscular contraction gradually subsided as the entire volume was administered. And the ‘donut sign’ was observed in the US short‐axis image; the sciatic nerve was visibly separated from the muscle fascia and circumferentially surrounded by the spread of LDC (Echeverry et al., 2010) (Figure 4). Circumferential was defined as injected LDC visualized in at least ≥75% surrounding the sciatic nerve short‐axis image (Brull, Macfarlane, Parrington, Koshkin, & Chan, 2011).

We considered these phenomena (hindlimb movement, disappearance of the movement and ‘donut sign’ on the US image after the LA injections) as a successful block.

In US short‐axis images, the lengths of long and short axes of sciatic nerve were measured. Mean distance from ellipse centres to distal femurs, and to skin were also measured.

## 3 RESULTS

### 3.1 Anatomical study in blood‐washed out porcine model

**FIGURE 5:**
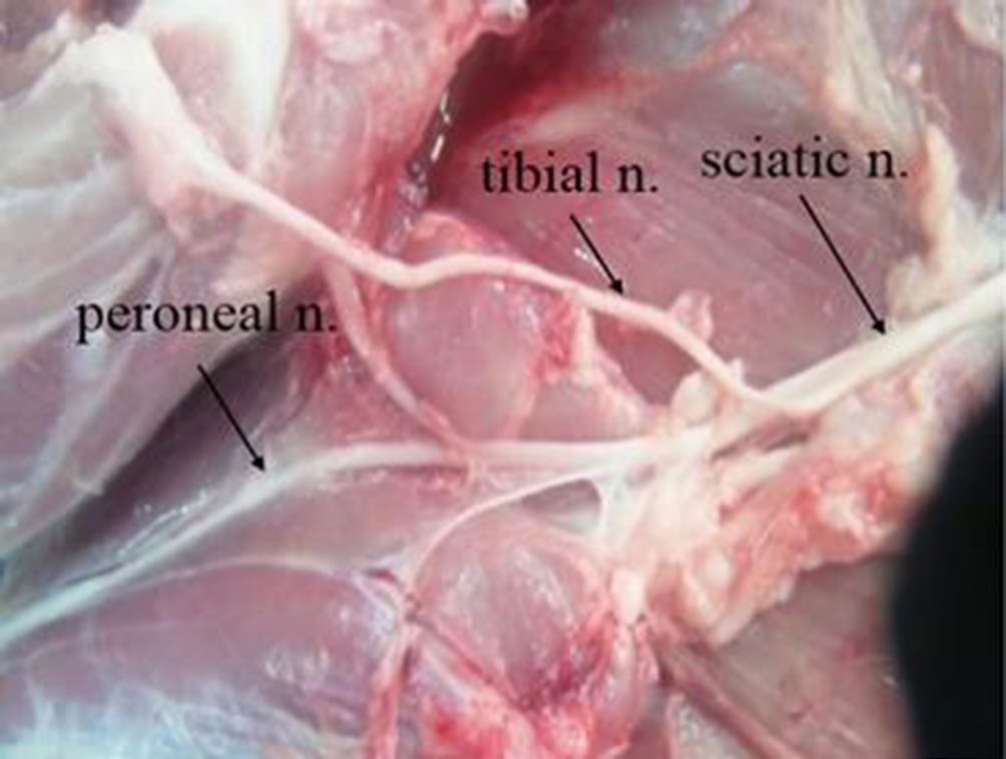
The dissected sciatic nerve and its branches. The sciatic nerve divided into the tibial and common peroneal nerve

The sciatic nerve and its branches were easily detected with reflecting the biceps femoris muscle in all cases (Figure 5). The tibial nerve was detected in the medial portion, and the common peroneal nerve in the lateral portion of posterior hindlimbs.

On H&E staining the sciatic nerve appeared as a large nerve bundle, and in cross sections methylene blue was observed outside the external epineurium (Figure 2). Gaps expanded between the nerve bundles by LDC were observed and no signs of inflammation were found.

### 3.2 Main US‐guided nerve blockade in live porcine model

US probes were located at about 6 cm from the patella crease level; immediately proximal to the bifurcation of the sciatic nerve into the tibial and common peroneal nerves were easily accessed by US also (Figure 3). The sciatic nerve was easily identified in positions mentions above in all cases.

**FIGURE 6:**
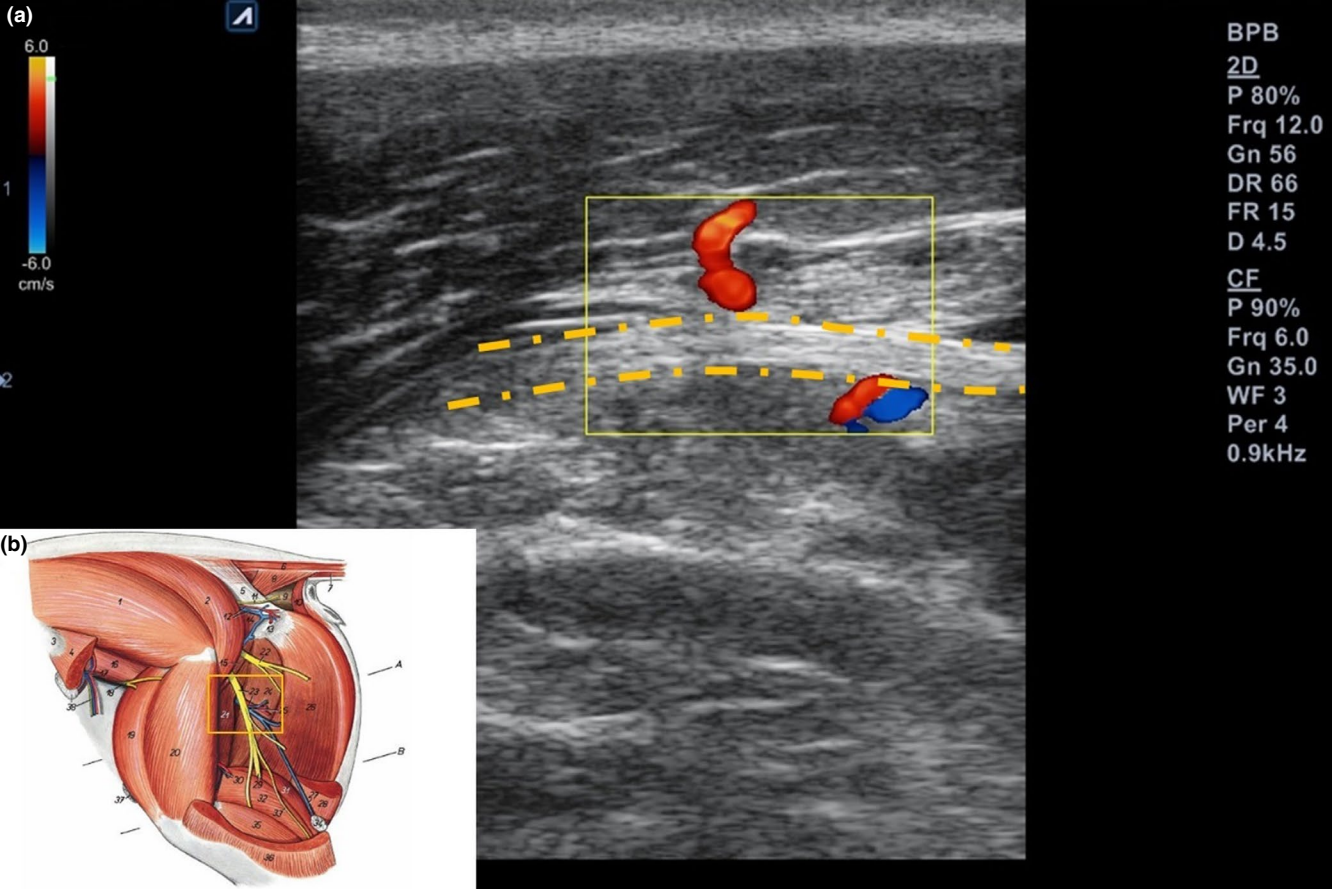
Ultrasound longitudinal image of the sciatic nerve (a) An inconsistent blood perfusion detected near the sciatic nerve (b) perfusion assumed a branch of the medial circumflex femoral artery according to an atlas of swine anatomy (Peter, 2012)

The distal femur was determined to use as the landmark because there were no vessels or other nerves surrounding the sciatic nerve (Figure 4). In 12.5% (2 of 16 nerves), very weak blood perfusion from a branch of the medial circumflex femoral artery crossing the sciatic nerve was detected about 6 cm proximal to the popliteal crease (Re et al., 2014) (Figure 6). However, because of its inconsistency, it could not be used as a landmark, and the diameter of the sciatic nerve was small at the midthigh level.

In US short‐axis images, the needle‐tip was positioned less than 1 cm lateral from the distal femur and about 2 cm deep to skin (Figure 4). Sciatic nerves had ellipse‐like cross sections with a long‐axis diameter of 4.8 ± 0.8 mm and short‐axis diameter of 2.8 ± 0.8 mm. The nerve was superficially located at 19.8 ± 1.4 mm beneath the skin and 3.4 ± 0.9 mm lateral to the distal femur.

The movement of the hindlimb concomitant with nerve stimulating and the ‘donut sign’ in US images after the LDC injection was confirmed in all 16 cases (100%).

## 4 DISCUSSION

We first described a method of performing US‐guided sciatic nerve block at midthigh level in a porcine model.

There have been some studies about US‐guided sciatic nerve block in dogs and calves. They performed sciatic nerve block via parasacral or transgluteal approach, in which the needle has to be advanced deeper than via the midthigh level (Benigni et al., 2008; Costa‐Farré et al., 2011; Echeverry et al., 2010; Re et al., 2014). Unlike those approaches, midthigh approach is easily accessible and has been shown better efficiency in blocking because of its anatomical peculiarities; such as superficial locations and the lack of other nerves and vascularity within the area (Echeverry et al., 2010; Re et al., 2014).

No vascularity in surrounding area can provide safer nerve block, but it can also contribute to difficulty in locating the sciatic nerve in US image because of the its lack of vascular landmarks (Barrington et al., 2008). In human, the popliteal artery runs parallel to the sciatic nerve in the popliteal fossa, and thus, the popliteal artery can be used as a landmark for popliteal sciatic nerve block. In pigs, there is no artery travelling parallel to the sciatic nerve. But weak perfusion was shown inconsistently, so colour image must be checked before the sciatic nerve block in midthigh level.

In this study, we used combined US and nerve stimulator to confirm the sciatic nerve. US guidance only can be useful for direct visualization of the nerves and their associated structures (Echeverry et al., 2010), but the structures closely attach to the nerve, muscular fascial planes, ligaments, and tendons can be misinterpreted due to similar echogenic shapes (Costa‐Farré et al., 2011). Vassiliou *et al*. reported the combined use of US and nerve stimulator provided better needle‐tip positioning for peripheral regional anesthesia than US alone in a porcine model (Vassiliou et al., 2012). But the clinical expertise with US technique does not seem to have advantages with using the nerve stimulator (Costa‐Farré et al., 2011).

We adopted an out‐of‐plane approach parallel to the sciatic nerve like Costa‐Farré et al's nerve block study in dogs (Costa‐Farré et al., 2011). The advantage of the out‐of‐plane approach is that the needle direction can be placed more parallel to the long axis of the nerve and that the needle insertion path is short (Costa‐Farré et al., 2011; Echeverry et al., 2010).

Most US‐guided peripheral nerve blocks have been performed using an in‐plane approach which has better needle visualization via real‐time inspection of the needle shaft and tip (Chin, Perlas, Chan, & Brull, 2008; Ruiz et al., 2014). But this approach is not always possible. Even with the clear US image, the needle advancement can be difficult, particularly in cases that require steep needle trajectories. In such cases, out‐of‐plane approach is very useful (Costa‐Farré et al., 2011; Fredrickson, Ball, & Dalgleish, 2011; Neice & Forton, 2018).

In our study, we tried out‐of‐plane approach according to needling in first anatomical study, thus in‐plane approach can be possible in this model also.

This is a descriptive study of the US‐guided midthigh sciatic nerve block technique using a porcine model. Royal et al. placed a catheter near the sciatic nerve at the parasacral level under US guidance in a pig after experimental femur fracture surgery (Royal et al., 2013). Treated pigs ate food, defecated earlier and obtained the beneficial analgesic effect. Therefore, subsequent studies to evaluate the analgesic effect of this approach are necessary and can be expected.

In conclusion, US‐guided midthigh sciatic nerve block can be very useful and easily performed in porcine models. In this report, we described the relation between the anatomical location and the US approach to the midthigh sciatic nerve in a porcine model from a clinical perspective.

## CONFLICT OF INTEREST

The authors declare no conflict of interest.

## AUTHOR CONTRIBUTION

**Mi Geum Lee:** Writing‐original draft; Writing‐review & editing. **Sung Uk Choi:** Data curation; Investigation. **Jae Kwan Lim:** Methodology. **Mee Ju Lee:** Methodology. **Ji Su Hong:** Methodology. **Mi Ok Baek:** Methodology. **Seung Zhoo Yoon:** Methodology. **Hee Yeon Park:** Conceptualization; Supervision. **Hyeon Ju Shin:** Conceptualization; Investigation; Methodology; Supervision; Writing‐original draft.

## ETHICAL STATEMENT

The authors confirm that the ethical policies of the journal, as noted on the journal's author guidelines page, have been adhered to and the appropriate ethical review committee approval has been received.

## Supporting information

Video S1
